# The coding mitochondrial genome of crayfish *Cambarellus patzcuarensis* (Cambaridae, Decapoda) with phylogenetic analysis

**DOI:** 10.1080/23802359.2025.2519197

**Published:** 2025-06-18

**Authors:** Chao Peng, Xianzhen Luo, Gaojie Chen, Linwen Xie, Peirong Ye, Sigang Fan

**Affiliations:** a College of Life and Environmental Sciences, Zoology Key Laboratory of Hunan Higher Education, Hunan Provincial Collaborative Innovation Center for Efficient and Health Production of Fisheries, Changde Key Innovation Team for Wetland Biology and Environmental Ecology, Hunan Provincial Key Laboratory for Health Aquaculture and Product Processing in Dongting Lake Area, Hunan Provincial Key Laboratory for Molecular Immunity Technology of Aquatic Animal Diseases, Hunan Engineering Research Center of Aquatic Organism Resources and Environmental Ecology, Changde Research Center for Agricultural Biomacromolecule, Hunan University of Arts and Science, Changde, China; b Key Laboratory of Aquatic Product Processing, Ministry of Agriculture, South China Sea Fisheries Research Institute, Chinese Academy of Fishery Sciences, Guangzhou, China

**Keywords:** Coding mitogenome, *Cambarellus patzcuarensis*, phylogeny, high-throughput sequencing

## Abstract

*Cambarellus patzcuarensis* is a popular aquarium freshwater crayfish in China. In this study, the mitochondrial genome of *C. patzcuarensis* was sequenced using Illumina HiSeq. The coding mitogenome of *C. patzcuarensis* is 15,000 bp in length, consisting 13 protein-coding genes (PCGs), 22 transfer RNAs, and two ribosomal RNAs. The A + T content was 70.17%. Ten PCGs had ATN as the start codon. Three PCGs were stopped with incomplete stop codon. The phylogenetic analysis shown that *C. patzcuarensis* was grouped with other *Procambarus* species. These results are useful for understanding the phylogenetic relationships of Cambaridae crayfish.

## Introduction

The Mexican dwarf crayfish *Cambarellus patzcuarensis* (Penn, 1953) is one of the crustaceans belonging to Cambaridae. It always inhabits swamps, streams, rivers, and ponds. Due to its small size, bright colors and ease of rearing, *C. patzcuarensis* has become a popular ornamental species in the world (Karadal and Türkmen 2017; Mojžišová et al. 2024). The body color of *C. patzcuarensis* is mostly brown. To date, the research of *C. patzcuarensis* was limited. The ultrastructure of the antennae and sensory hairs of *C. patzcuarensis* was reported by Kor et al. (2023). The most preferred substrate for *C. patzcuarensis* was basalt (Karadal and Türkmen 2017). Mitochondrial DNA is an effective genetic marker for population genetics and molecular phylogenetics (Desalle et al. 2017). Nowadays, only partial sequences of 16S, 12S, and *COX1* from *C. patzcuarensis* mitochondrial DNA sequences were shown in GenBank, which is significantly insufficient for evolution and conservation genetics of *C. patzcuarensis.* The coding mitochondrial genome (mitogenome) of *C. patzcuarensis* is an essential resource for evolutionary research. In the present study, the coding mitogenome of *C. patzcuarensis* was sequenced with high-throughput sequencing and characterized for the first time, which provided genetic resources for its phylogenetic study.

## Materials and methods

**Figure 1.**
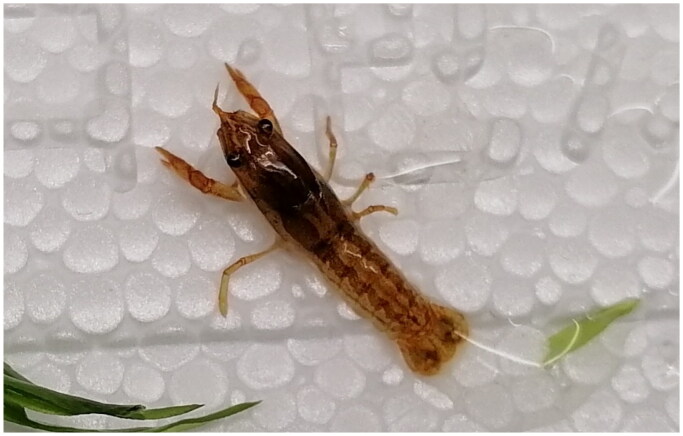
Representative of *C. patzcuarensis* collected in the study (photograph by Sigang Fan).

A live shrimp used in this study was obtained from Beijiang River, Qingyuan, Guangdong, China (23°18′ N, 113°17′ E) (Figure 1). Based on its morphology, especially the partial 16S (MF449471.1), 12S (JX127602.1), and *COX1* (JX127894.1) sequences of *C. patzcuarensis* had more than 99% identity with that of the shrimp. Therefore, the shrimp obtained by us should be *C. patzcuarensis*. It was transported and deposited in the South China Sea Fisheries Research Institute (contact person: Sigang Fan, email: fansigang@scsfri.ac.cn) with voucher number BJ150068. The muscle was sampled and frozen into liquid nitrogen rapidly. Total genomic DNA was extracted using a TIANamp Marine Animals DNA Kit (Tiangen, Beijing, China), according to the manufacturer’s protocol. DNA libraries were generated using the Hieff NGS^®^ MaxUp II DNA Library Prep Kit for Illumina^®^ (Yeasen, Shanghai, China) and sequenced using Illumina HiSeq 2000 (Illumina Inc., San Diego, CA) by Sangon Biotech Co., Ltd. (Shanghai, China). After removing the adapter sequences, poly-N and low quality bases (base quality ≤20) from raw data by Fastp 0.17.0 (Chen et al. 2018), the clean reads were assembled with SPAdes v3.15.2 and PRICE (Bankevich et al. 2012; Ruby et al. 2013). Transfer RNA (tRNA), protein-coding genes (PCGs), and ribosomal RNA (rRNA) were annotated and analyzed by MITO Web Server 2 (http://mitos2.bioinf.uni-leipzig.de/index.py) (Bernt et al. 2013). A circular mitochondrial genome map was drawn using OGDRAW v1.3.1 (Greiner et al. 2019). The phylogenetic analyses were conducted based on concatenated 13 PCGs of *C. patzcuarensis* and other 14 species of Cambaridae downloaded from GenBank. *Homarus americanus* was selected as an outgroup (Kim et al. 2011). Phylogenetic relationship was reconstructed using maximum-likelihood (ML) with Jones–Taylor–Thornton (JTT) model in MEGA 7.0.26 (Kumar et al. 2018). The reliability of the tree topology was evaluated using bootstrap support with 1000 replicates. The read coverage depth map of *C. patzcuarensis* is shown in Supplementary Figure S1.

## Results

**Figure 2.**
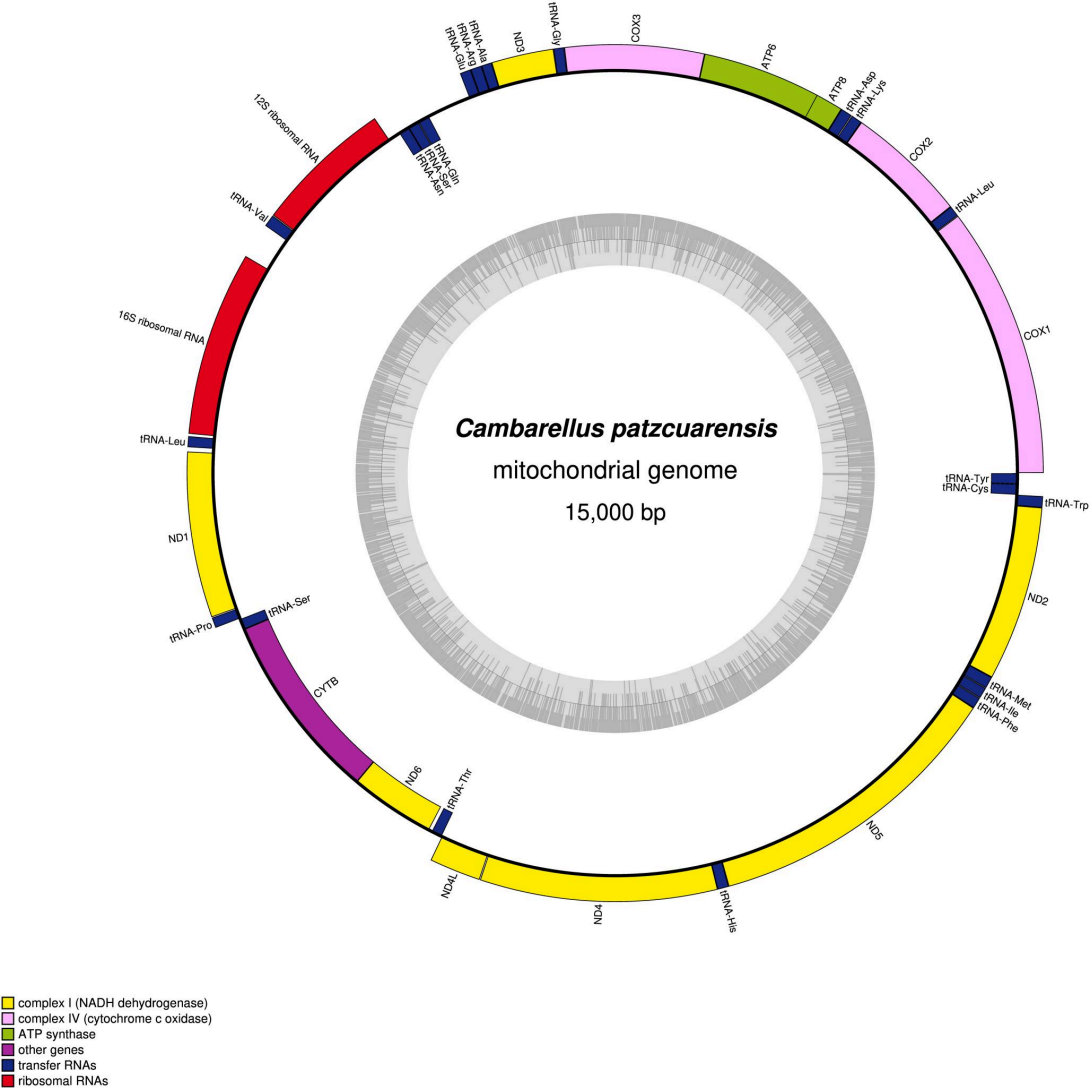
Circular maps of the mitochondrial genome of *C. patzcuarensis*. Genes on the outside and inside of the circle are transcribed in the clockwise and counterclockwise directions, respectively. The dark and light gray bars in the inner circle denote G + C and A + T contents, respectively.

**Figure 3.**
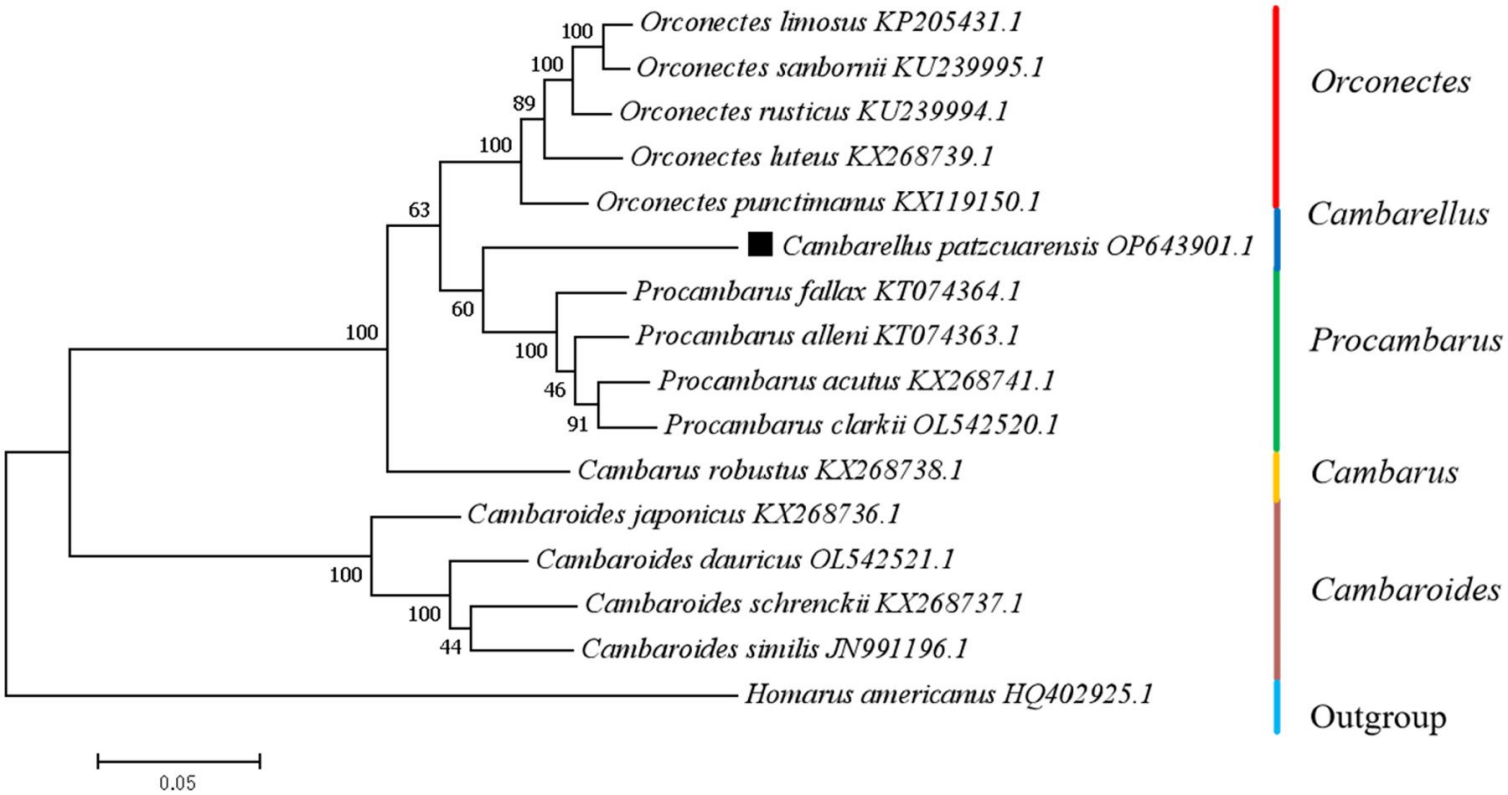
Phylogenetic tree of *C. patzcuarensis* and related species based on maximum-likelihood (ML) method. Bootstrap support values are indicated at each node. Accession numbers are indicated after the species names. *C. patzcuarensis* was indicated with a black square. The following sequences were used: *Orconectes limosus* (KP205431.1) (Gan et al. 2016), *Orconectes sanbornii* (KU239995.1), *Orconectes rusticus* (KU239994.1), *Orconectes luteus* (KX268739.1), *Orconectes punctimanus* (KX119150.1), *Cambarellus patzcuarensis* (OP643901.1), *Procambarus fallax* (KT074364.1), *Procambarus alleni* (KT074363.1), *Procambarus acutus* (KX268741.1), *Procambarus clarkii* (OL542520.1) (Luo et al. 2023), *Cambarus robustus* (KX268738.1), *Cambaroides japonicus* (KX268736.1), *Cambaroides dauricus* (OL542521.1) (Luo et al. 2023), *Cambaroides schrenckii* (KX268737.1), *Cambaroides similis* (JN991196.1) (Kim et al. 2012), and *Homarus americanus* (HQ402925.1) (Kim et al. 2011).

The double-stranded circular mitogenome of *C. patzcuarensis* was 15,000 bp in size (GenBank accession number: OP643901.1), with the A + T content of 70.17% (29.80% A, 19.41% G, 40.37% T, and 10.42% C). It consisted of 13 PCGs, two rRNA genes, and 22 tRNA genes (Figure 2, Supplementary Table S1). Of the 37 genes, 28 were encoded by the heavy strand. Remaining nine genes including two PCGs (*CYTB* and *ND6*) and seven tRNA genes were encoded by the light strand (Figure 2; Table S1). *COX2*, *ND5*, and *ND2* were initiated with GTG, and another PCGs were started with ATN (Table S1). Ten PCGs contained TAN as the stop codon except *COX2*, *CYTB*, and *ND5* (stopped with incomplete T– stop codon). 16S rRNA and 12S rRNA were 1048 bp (73.47% AT content) and 787 bp (73.32% AT content) in length, respectively. All tRNA genes ranged from 59 to 70 bp in size (Table S1). D-loop was 265 bp length and located between tRNA-Glu and tRNA-Gln (Table S1). The phylogenic relationship among species from five genera of Cambaridae was chosen and analyzed. Each species from the same genus was grouped in a branch. *Procambarus* and *Cambarellus* were in sister relationship, and then clustered with *Orconectes* (Figure 3).

## Discussion and conclusions

In this study, the coding mitochondrial genome of *C. patzcuarensis* was first sequenced, analyzed, and reported in genus *Cambarellus*, which was provided valuable resource to reveal the phylogenetic relationship of *Cambarellus* with other genus in Cambaridae. The A + T content of *C. patzcuarensis* was similar with *P. acutus* (72.10%) and higher than *P. alleni* (67.98%) and *P. fallax* (67.16%). The gene order of *C. patzcuarensis* mitogenome was the same distinctive arrangement found in other *Procambarus* species (Kim et al. 2012; Luo et al. 2023). The D-loop sequence is too short and may be incomplete. This phenomenon has also been observed in *Procambarus alleni*, *Procambarus fallax*, and *Pontastacus leptodactylus* (Alvanou et al. 2023). Due to high AT content and variability, D-loop is difficult to assemble in freshwater crayfish. The results of phylogenetic relationships were similar with previous reports (Luo et al. 2023). Based on the low bootstrap supports (60 and 63), *C. patzcuarensis* may be either basal to species of *Procambarus* or *Orconectes* (Figure 3). The relationship between *C. patzcuarensis* and species from genus *Procambarus* and *Orconectes* were not clear. More related taxa are need to elucidate the relationship between *C. patzcuarensis* and species from genus *Procambarus* and *Orconectes*.

In conclusion, our study first presented the coding mitogenome of *C. patzcuarensis* in *Cambarellus*, which could provide insight into the molecular evolution and conservation of this species and *Cambarellus*.

## Supplementary Material

Supplemental Material

## Data Availability

The data that support the findings of this study are openly available in GenBank of NCBI under the accession no. OP643901.1. The associated BioProject, SRA, and Biosample numbers are PRJNA1079438, SRR28058353, and SAMN40043099, respectively.
