# Supplementary material for: The coding mitochondrial genome of crayfish Cambarellus patzcuarensis (Cambaridae, Decapoda) with phylogenetic analysis
Source: Mitochondrial DNA B Resour. 2025 Jun 18;10(7):602–5. doi: 10.1080/23802359.2025.2519197 (PMC12180320; doi:10.1080/23802359.2025.2519197)
Supplement: Supplemental Material [file TMDN_A_2519197_SM7211.docx]

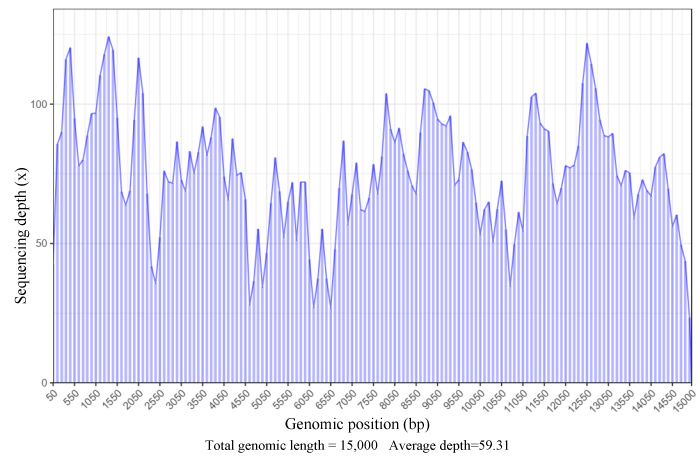


Figure S1. Sequencing depth and coverage map of *C. patzcuarensis.*

Table S1. List of annotated mitochondrial genes of *C. patzcuarensis*

| Gene | Strand | Sequence Location | Size | Start codon | Stop codon | Intergenic region |
| --- | --- | --- | --- | --- | --- | --- |
| COX1 | + | 1-1536 | 1536 | ATG | TAA | 1 |
| tRNA-Leu2 | + | 1538-1600 | 63 |  |  | 0 |
| COX2 | + | 1601-2288 | 688 | GTG | T-- | 0 |
| tRNA-Lys | + | 2289-2352 | 64 |  |  | 2 |
| tRNA-Asp | + | 2355-2420 | 66 |  |  | 0 |
| ATP8 | + | 2421-2579 | 159 | ATG | TAG | -7 |
| ATP6 | + | 2573-3247 | 675 | ATG | TAA | -1 |
| COX3 | + | 3247-4035 | 789 | ATG | TAA | 0 |
| tRNA-Gly | + | 4036-4095 | 60 |  |  | 0 |
| ND3 | + | 4096-4449 | 354 | ATT | TAG | 0 |
| tRNA-Ala | + | 4450-4508 | 59 |  |  | 0 |
| tRNA-Arg | + | 4509-4572 | 64 |  |  | 0 |
| tRNA-Glu | + | 4573-4635 | 63 |  |  | 0 |
| Partial D-loop |  | 4636-4900 | 265 |  |  | 0 |
| tRNA-Gln | - | 4901-4969 | 69 |  |  | 1 |
| tRNA-Ser1 | - | 4971-5037 | 67 |  |  | 0 |
| tRNA-Asn | - | 5038-5101 | 64 |  |  | 71 |
| 12S ribosomal RNA | + | 5173-5959 | 787 |  |  | 2 |
| tRNA-Val | + | 5962-6031 | 70 |  |  | 201 |
| 16S ribosomal RNA | + | 6233-7280 | 1048 |  |  | 13 |
| tRNA-Leu1 | + | 7294-7355 | 62 |  |  | 24 |
| ND1 | + | 7380-8318 | 939 | ATG | TAA | 3 |
| tRNA-Pro | + | 8322-8381 | 60 |  |  | 4 |
| tRNA-Ser2 | - | 8386-8450 | 65 |  |  | 0 |
| CYTB | - | 8451-9585 | 1135 | ATG | T-- | -1 |
| ND6 | - | 9585-10100 | 516 | ATT | TAA | 19 |
| tRNA-Thr | - | 10120-10184 | 65 |  |  | 2 |
| ND4L | + | 10187-10480 | 294 | ATG | TAA | 3 |
| ND4 | + | 10484-11827 | 1344 | ATG | TAA | -1 |
| tRNA-His | + | 11827-11890 | 64 |  |  | 0 |
| ND5 | + | 11891-13616 | 1726 | GTG | T-- | 0 |
| tRNA-Phe | + | 13617-13680 | 64 |  |  | 1 |
| tRNA-Ile | + | 13682-13745 | 64 |  |  | 1 |
| tRNA-Met | + | 13747-13814 | 68 |  |  | 0 |
| ND2 | + | 13815-14807 | 993 | GTG | TAA | 0 |
| tRNA-Trp | + | 14808-14871 | 64 |  |  | 0 |
| tRNA-Cys | - | 14872-14935 | 64 |  |  | 0 |
| tRNA-Tyr | - | 14936-14999 | 64 |  |  | 0 |
